# Integration of nematology as a training and research discipline in sub-Saharan Africa: progress and prospects

**DOI:** 10.1163/15685411-00003291

**Published:** 2019-10-22

**Authors:** Laura Cortada, Inge Dehennin, Wim Bert, Danny Coyne

**Affiliations:** 1 International Institute of Tropical Agriculture (IITA), Kasarani, P.O. Box 30772-00100, Nairobi, Kenya; 2 Nematology Research Unit, Faculty of Sciences, Ghent University, K.L. Ledeganckstraat 35, 9000 Ghent, Belgium

**Keywords:** curricula, capacity building, education, EUMAINE, extension services, IMaNema, PINC, tertiary institutions, universities

## Abstract

Within sub-Saharan Africa (SSA), although nematodes are viewed among the most important threats to crop production and food security, the presence of trained nematologists working within this discipline has traditionally been viewed as scarce. The few research studies concerning this subject address this topic from a country or sub-regional perspective and generally portray nematology as ‘insufficient’. Over the past two decades, a few initiatives have been instrumental in building greater nematology expertise. For the first time a structured survey was undertaken, involving interviews with individuals from SSA that were (or currently are) involved in nematology training programmes, research, national extension services or in African universities. This paper provides evidence of the positive impact of various initiatives and shows an increase in the number of available nematology positions, together with high rates of graduates that return home to occupy qualified positions. Our findings will help researchers, policy makers and donors to identify areas requiring support to increase the promotion of nematology in SSA and to make an impact for end-users.

Looking back over the history of nematology, it is striking how concealed a discipline it has remained and how recent a scientific discipline it is, astoundingly so in Africa compared to other parts of the world. For example, although the first reports of nematology from the whole of Africa south of the Sahara was published in 1904 regarding root-knot nematodes, with other notes on other nematode species appearing later (Corbett, 1967; Meyer, 2017), it was not until the 1950s onwards that any significant reports were made with respect to nematology in Africa (Merny, 1966; Caveness, 1992). Even then, such reporting was limited and stemmed from just a small number of individuals. Historically, it is clear that the recognition and the attention paid to nematology in the plant protection sector has been far less than for other disciplines (*i.e.*, entomology) and, as a consequence, fewer professionals have focused their careers on this fascinating research subject (Sharma *et al.*, 1997). It may be argued, however, that the lack of attraction to nematology has been the result of limited exposure to the discipline in general. We would like to show that, through a combination of greater exposure and a more facilitatory environment, nematology, as a discipline, can be made more attractive to future generations of researchers. Furthermore, it is the aim of this article to show that under such a harmonious environment, current attitudes and perceptions of nematology can be fundamentally altered.

Given the tremendous actual and potential impact that plant-parasitic nematodes (PPN) can have on African agriculture, it is surprising that awareness and understanding of nematology have remained almost unchanged since the second half of the 20th century. In 1967, Corbett stated that “ignorance on the part of the farmer, on the part of the extension worker, of the agricultural administrator and even of the nematologist” were obstacles that nematology had to overcome as a field of study in Africa. However, PPN remain a persistently overlooked concern, re-sulting in crop losses that can amount to billions of dollars (Coyne *et al.*, 2018). In the current scenario, where Corbett’s challenges persist, the need to increase agricultural productivity and produce safer food in Africa is more critical than ever. According to the United Nations Statistical Division (UNSD), the global population will increase by 2.2 billion people by 2050, with a significant portion of this growth in sub-Saharan Africa (SSA) (UNSD, 2017). To ensure food security, agricultural productivity in SSA needs to more than double within the next two decades (FAO, 2017). To meet this challenge, agricultural systems need to shift substantially and intensify. As witnessed in the Asian Green Revolution, such a change brings with it a maelstrom of pest and disease issues (Hazell, 2009). However, unlike in Asia, any revolution in Africa will need to consider a new set of tools to deal with any such pestilence, or rather a lack of tools, as the withdrawal from the market of numerous Class I synthetic chemical pesticides has transformed the situation. Many of these banned pesticides were nematicides, which paradoxically further compounds the nematode problem: not only is there too little awareness regarding PPN, they are also often overlooked and misdiagnosed. Furthermore, the most effective tools previously relied upon to manage them are now obsolete, requiring alternatives to be sought. Therefore, there is currently a pressing need for nematologists to create awareness, provide advice and conduct research for new management options. Addressing nematology is an integral component of soil health and a critical element to ensure food safety, both now and for future generations.

Over the past two decades, a few initiatives have been instigated in SSA towards enhancing nematology capacity. The Nematology Initiative for Eastern and Southern Africa (NIESA) (Gatsby Charitable Foundation, 2014) was a particularly prominent effort from 2004 to 2011, established as a North-South cooperation programme to boost regional capacity, stakeholder awareness and research on plant-parasitic nematology. It was led by Rothamsted Research (UK), CABI Bioscience (UK) and Reading University (UK), with a strong participation from active nematologists within the Eastern and Southern Africa region. This initiative focused primarily on tertiary education through training at M.Sc. and Ph.D. level, as well as equipping nematology laboratories to provide platforms for continuity of newly-trained nematologists. It also helped to expand the nematology network in Africa, through links with the Nematological Society of South Africa (NSSA). Another initiative that has perhaps gained the greatest recognition for building nematology capacity across Africa (and beyond) has been the international M.Sc. nematology programme from Ghent University (UGent) sponsored by the Flemish Interuniversity Council – University Development Cooperation (VLIR-UOS), which has provided a wealth of scholarships to Africa since 1992 throughout its various phases (Postgraduate International Nematology Course (PINC), European Master of Science in Nematology (EUMAINE) and the present International Master in Nematology (IMaNema)). Finally, it is relevant to mention the International Institute of Tropical Agriculture (IITA), which has effectively delivered approximately 50 years of nematology research and training to SSA, transforming IITA into the lead centre of the Consultative Group for International Agricultural Research (CGIAR) in terms of nematology expertise (Karamura *et al.*, 1996; Sharma *et al.*, 1997).

Despite these efforts having an undeniable impact on the field of nematology in SSA, we are yet to understand fully the actual impact of such initiatives on the development of nematology as a science, and their spill-over effect to small-holder farmers in SSA. Even within our discipline, there is a general perception that few people ultimately pursue nematology as a career in SSA and that, upon accessing international training programmes, trainees fail to return, thus ‘brain draining’ Africa of nematology capacity. It could be argued that the sheer geographic coverage of the SSA region, the historical, cultural and linguistic heterogeneity, or the lack of political and economic stability, are key determinants for the lack of sustained nematology in SSA. Understandably, such numerous factors play a role, as they do for most other such scientific disciplines. However, there is no denying that a scarcity of nematology know-how by individuals at key decision-making levels creates voids for generating nematology-related positions or activities, whether this be within national research programmes or in academic institutes. We could suggest, therefore, that insufficient local expertise is a constraining factor, which is compounded by the limited available information regarding this subject in SSA. The reference to ‘insufficient’ nematologists in SSA in the scarce available literature has been attributed to an overarching lack of funding and capacity across public and private sectors, from academia to national extension systems (Abebe *et al.*, 2015; Talwana *et al.*, 2015). Yet, when embarking on this line of research, we were unable to establish any information reporting on systematic, specific or even broad data collection and analysis regarding the presence of SSA students in (inter)national nematology training programmes, the role of the local research organisations in the support and promotion of this subject, or the inclusion of nematology in the curriculum of the universities in a broader geographic context for the SSA region.

Therefore, to assess the chief obstacles to the advance of nematology within the region, it would be crucial to gather, assess and analyse this information critically. The main objectives of our study were to understand better: *i*) which institutions are providing nematology training to SSA students; *ii*) in which countries and organisations trainees develop their careers; *iii*) accessibility to nematology-related positions by trainees; *iv*) the return rate of African nematologists following postgraduate studies outside the continent; and *v*) the potential for the development of nematology as a science in SSA in light of *i*-*iv*. To our knowledge, this is the first time that structured interviews have been undertaken to collate this information and to provide gender-disaggregated data related to nematology trainees/professionals from SSA, which presents an assessment over the past 25 years.

The main findings of our study indicate that, despite nematology being a little-known discipline in SSA, remarkable progress and increased awareness has occurred over the last two decades, allowing more professionals to return to SSA to occupy qualified positions in academia, and in public or private institutions. We expect that our results will help policy makers, national agricultural and research programmes, academics and donors to identify the strengths, the weaknesses and the opportunities ahead of us to promote nematology further in the region.

We believe there is light at the end of this long tunnel – a light that is quite possibly brighter than we previously anticipated.

## Materials and methods

Qualitative and quantitative data were collected using a range of tools, including online questionnaires, key informant interviews, analyses of students’ repositories from academic institutions, and a desktop literature review. A structured questionnaire (see Table S1 in the online edition of this journal, which can be accessed *via* brill.com/nemy) was distributed using an online platform (SurveyMonkey, San Mateo, CA, USA; www.surveymonkey.com); interviews were conducted from October to November 2017 to graduates and current SSA students attending IMaNema, graduates from the former PINC and EUMAINE, lecturers from SSA universities, nematologists in SSA research centres, extension workers in national agricultural programmes, and members from the Nigerian Society of Nematologists (NISON). South Africa was purposely omitted from this survey as the status of nematology in the country has already been exhaustively documented (Fourie *et al.*, 2017).

The survey had nine introductory questions, which then guided interviewees into two separate tracks. Track A “Scholarly/scientific career-oriented path” was designed to target interviewees who had been able to pursue a scientific and/or academic path in nematology and who remain involved; respondents that did not fit this profile were redirected to Track B “Non-research careeroriented path”, designed to capture the views of those who developed their careers away from science/universities. Both pathways included open-ended and closed questions (either with single or multiple response options). Towards the end, the tracks converged into two questions to capture the personal perceptions of all interviewees on the overall status of nematology in Africa; the information derived from these was developed into a word-cloud (WordClouds; https://www.wordclouds.com/). Key informant interviews (KII) were undertaken *via* email (see Table S1 in the online edition of this journal, which can be accessed *via* brill.com/nemy), and a focal group discussion (FGD) was organised at Kenyatta University in Nairobi, Kenya, on 12 January 2018 involving the participation of 66 people from the private, public and academic sectors; another FGD took place with seven staff members from five universities in Kenya (Kenyatta University, Moi University, Jomo Kenyatta University of Agriculture and Technology, University of Embu and the University of Nairobi) in order to gain an understanding of how nematology fits within the curriculum in the University system and on the current education policies at national level.

To identify the geographic origin of the graduates and obtain gender-disaggregated country profiles for SSA, we conducted an analysis of the PINC/EUMAINE/IMaNema database (1992-2017) regarding student application and graduation. Finally, the number of publications related to the study of PPN within the SSA region was determined through a filtered search on Scopus database (filtered search at Scopus Database as “plant parasitic nematodes” w/africa) (see Table S2 in the online edition of this journal, which can be accessed *via* brill.com/nemy).

## Results and discussion

### THE UGENT’S M.SC. PROGRAMMES: 25 YEARS OF LEGACY IN SUB-SAHARAN AFRICA

**Fig. 1:**
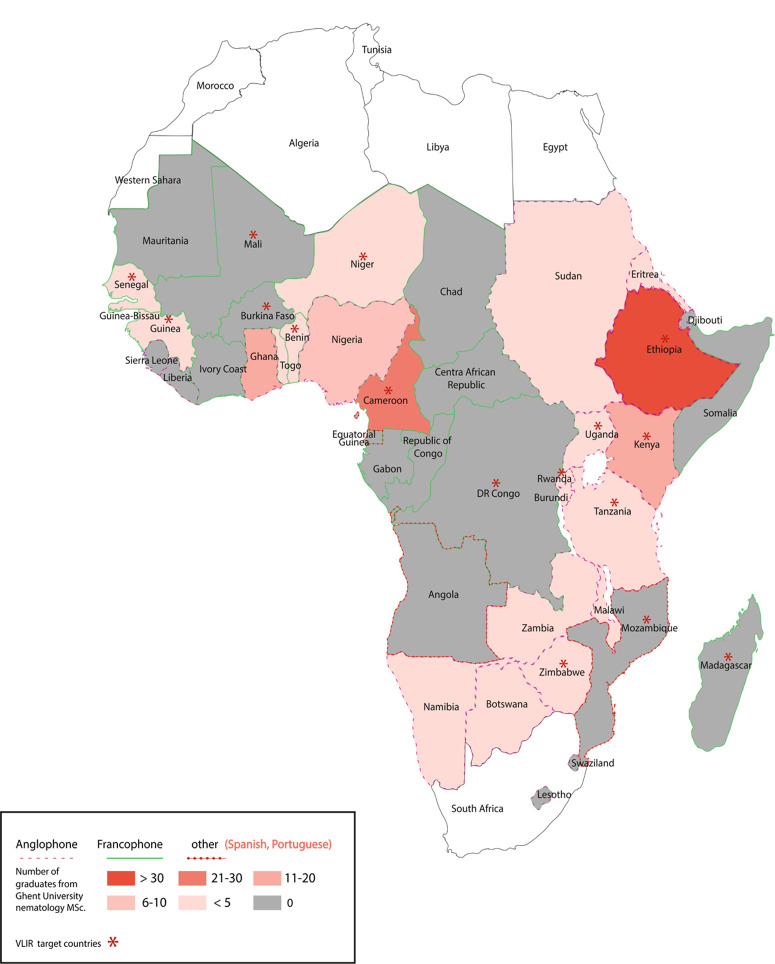
Country profiles from Ghent University database of alumni for the sub-Saharan Africa region, including information on VLIRUOS scholarships’ eligibility per country and local language. NB: Recipient countries such as Morocco and South Africa, which are included in the VLIR-UOS, are not considered in the SSA group, based on the classification of UN for sub-Saharan Africa (UN Statistics Division; Geographic Regions Africa: https://unstats.un.org/unsd/methodology/m49/).

In 1992, PINC started as a 1-year programme at UGent, aimed at providing international nematology education at the M.Sc. level, and it has since continued to the present day being the only M.Sc. in the world aimed at building capacity of our discipline. The M.Sc. covers a wide range of nematology disciplines, receiving the support from a corps of international lecturers, universities and organisations, to provide participants with an unparalleled access to a global nematology network and research. In 2007, PINC developed into a 2-year programme that ran alongside the EUMAINE programme from 2008 to 2014. EUMAINE was a 5-year project funded by the Culture and Audio-visual Executive Agency (EACEA) of the European Union (EU), to provide scholarships for both EU and non-EU countries. The programme was coordinated by UGent with participation from a consortium of EU universities and associated partners. The EUMAINE programme closed in 2015, but support for an adjusted PINC, currently named IMaNema M.Sc., was secured to continue the course, which currently stands as the only international M.Sc. programme specialised in nematology across the globe. Under the 2-year IMaNema programme, the existing collaborations with the EU universities and associated partners have been extended with new partnerships in Austria, Brazil, China, Ethiopia and Kenya. The programme has been continuously supported by the Belgian Government through the Agency for Belgian Development Cooperation (ABOS), and currently through the Flemish Interuniversity Council – University Development Cooperation (VLIR-UOS). Scholarships target graduate students from specific countries, currently including 17 from SSA (Fig. 1). UGent also provides additional grants for students from low income countries from the DAC list of ODA recipients to attend the international M.Sc. programme (UGent, 2017).

**Fig. 2:**
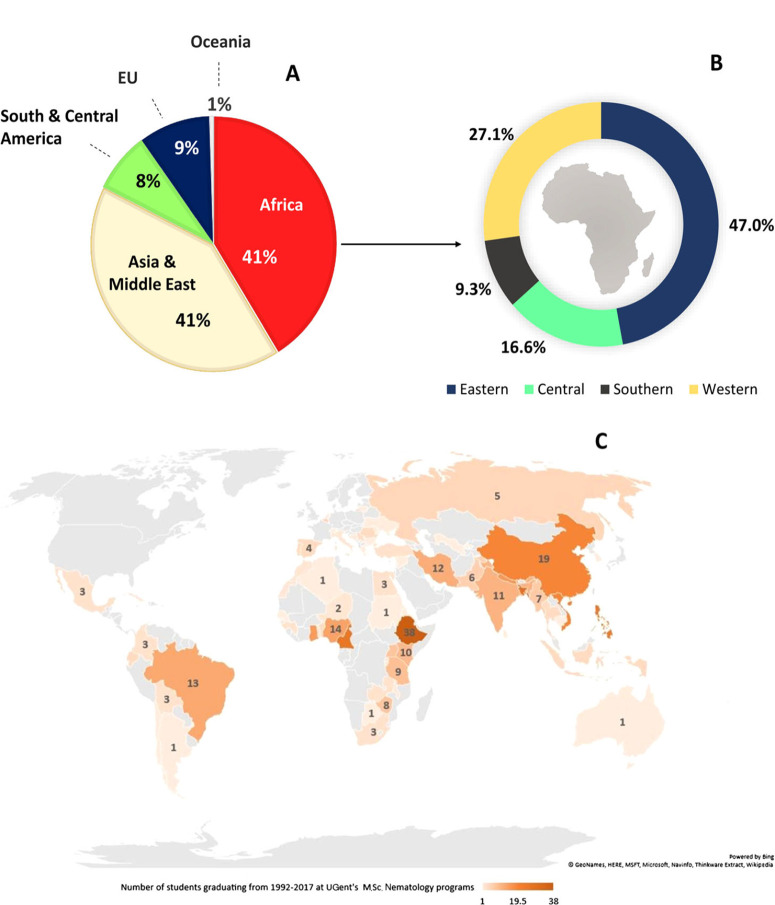
A: Percentage of students successfully graduating from the M.Sc. programmes (PINC, EUMAINE and IMaNema) of Ghent University (UGent) clustered by continent; B: Percentage of participants by region (Eastern, Southern (excluding South Africa), Central andWestern) in SSA; C: Global map with the countries that had women and men graduates in UGent’s Nematology M.Sc. programme.

Due to this unique long-standing tradition of nematology training by PINC, EUMAINE and IMaNema to international students, we considered that analysis of the UGent alumni database was particularly useful to determine the effect of such programmes on SSA over the last 25 years. Results indicate that between 1992 and 2017 a total of 391 students (162 women and 230 men) from 68 nationalities successfully graduated with a M.Sc. in nematology under the UGent programmes. Africa provided more students than any other continent, representing 25 countries with 161 students, or 41% of the total (Fig. 2A). Within Africa, 96% of students originated from SSA (excluding Southern Africa), representing 39% of total students graduating over two decades. However, within SSA (Fig. 2B), most students originate from Eastern Africa (eight countries, 47% graduates) and Western (seven countries, 27.2% graduates), followed by Southern (four countries, 9.3% graduates) and Central Africa (one country, 16.6% graduates). This latter region is represented by Cameroon only, with 25 graduates. Noteworthy, eight African countries appear in the top 15 of the world’s ranking in terms of number of students graduating (Fig. 2C).

The country profile of UGent’s database (Fig. 1) shows that nematology knowledge is mainly polarised in Western/ Central (Cameroon, Ghana, Nigeria) and Eastern Africa (Ethiopia and Kenya) with a significant gap in Central and Southern regions. Differences could quite possibly be attributed to the linguistic barriers, as a successful enrolment requires a strong command of English; overall, graduates from anglophone countries represent 72.4% of the total *vs* 27.2% from francophone regions and there were no lusophone students until 2017. Due to insufficient proficiency in English (a minimum of a B1 level of English is required) a large proportion of non-native English-speaking students fail to provide the required academic certificates for admission to UGent. The language distinction is possibly best highlighted using Cameroon as an example, where the tertiary education is largely in French (90.9%) (State University of Cameroon, 2012), but 76% of Cameroonian students attending the nematology courses were from two anglophone state universities (Buea and Bamenda Universities). Consequently, even if participants from specific non-English-speaking SSA countries are prioritised through the Belgium scholarships programme (Fig. 1), it is unlikely that the number of French-and Portuguese-speaking students will increase in the coming years. However, linguistic barriers should be consciously addressed as these could have major implications in the future cohorts of nematology SSA graduates. This is particularly relevant for the Central andWestern Africa francophone countries that are no longer eligible for VLIR-UOS scholarships, and from where there have been either no or very few participants (Fig. 1). Overcoming this challenge would require additional support to transform the academic curricula in these countries to improve the English proficiency of students, or to provide supporting teaching materials with more francophone and lusophone content.

**Fig. 3:**
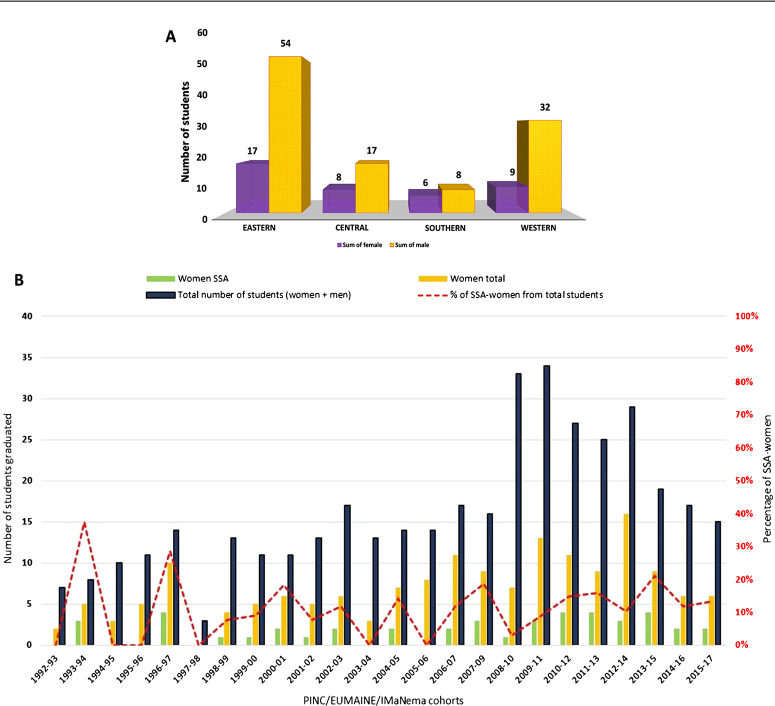
A: Number of women and men by each sub-Saharan Africa region (Eastern, Southern (excluding South Africa), Central and Western); B: Evolution of the number of women from SSA relative to the total number of women participating in the PINC, EUMAINE and IMaNema programmes from 1992 to 2017.

With respect to gender, a close inspection reveals important disparities between SSA and non-SSA regions. In SSA, the overall participation of women has been low compared to men (Fig. 3A), and in some instances there were no SSA female participants at all (2003-2004 and 2005-2006 cohorts). However, in Asia the proportion of female students was similar (42.2%) to male students, whilst in Europe and in South/Central America the promotion of female participation was found to be 60% or higher. The gender gap observed across the entire SSA region was more evident in Western Africa, where fewer than one in four graduated students were female (Fig. 3A), although striking differences were also encountered in some East Africa countries, such as Ethiopia, where just five women from a total of 38 Ethiopian participants graduated over the past 25 years; in some countries there were no female students at all (Burundi, Eritrea, Guinea, Niger, Senegal, Sudan, Togo and Zambia). Tanzania was the only country in SSA where more female students than males have graduated over the last 25 years. In part, the challenge also lies with the low number of female applicants: Ethiopia has the largest number of scholarship applicants, but only 7.4% are women (2008-2016 data). Despite the relatively low participation of women from SSA over the years, the trend does appear to be progressively changing, with increasing numbers attending (Fig. 3B). To correct this gender discrepancy, a positive bias has been semiformally applied to female applications from SSA for UGent scholarships. An obstacle to this has been experienced due to female applicants’ inability to meet the necessary academic criteria and thus fail to gain admission to the M.Sc. course. There are undoubtedly numerous reasons for this, including a reflection of the general bias traditionally held towards the prioritisation of males for education in SSA (FAWE, 2017). Such traditional barriers reduce the chances of females completing their primary education by 50% compared to their male contemporaries. In 2005, for example, there were 22 million deschooled girls in SSA (Save the Children, 2005) meaning that women comprised two-thirds of illiterate adults in SSA. In addition, on gaining access to higher education, female drop-out rates during the first year of university are disproportionately high, compared to males, with retention rates for women remaining low, especially in the absence of social protective networks (Egne, 2014). There are multiple reasons for this ultimately low representation of women in SSA academia, from general sociocultural attitudes, to discrimination, sexual harassment/violence from male colleagues, teachers’ insensitivity towards gender-related needs, lack of counselling, financial constraints or differences in their educational competencies prior to reaching tertiary education (Melese & Fenta, 2009; Mersha *et al.*, 2013; Molla&Cuthbert, 2014; O’Keeffe, 2017).

### VOICES FROM AFRICA: WHAT OUR NEMATOLOGY COLLEAGUES SHARED WITH US

#### Nematology within academia in sub-Saharan Africa

To understand how SSA students gained exposure to nematology, we asked at which stages of the tertiary education they received training. Respondents answered that they received training at the B.Sc. (19.8%), the M.Sc. (61.9%) and Ph.D. (18.3%) levels. In total, 124 respondents from 20 countries answered the MonkeySurvey^®^ corresponding to 34 women, 74 men and 16 undefined. In terms of age, 1% of the interviewees were younger than 25, 42% between 25 and 35, 42% between 36 and 45, and 29% were 46 or older. Regarding regions, Eastern and Western Africa had the most responses (62.4% and 32.8%, respectively). The highest academic qualification of interviewees was M.Sc. (55.3%), Ph.D. (39.5%) and B.Sc. (5.3%). These data correlate well with the academic level at which interviewees became involved in nematology research; relatively few were at the undergraduate level (12.6%), and most during their M.Sc. (56.3%) or Ph.D. (31.1%) studies. The data clearly indicate that there is lit-tle exposure to nematology at the undergraduate level. Despite the percentage participation in research at the higher education levels appearing high, the actual number of respondents that enrolled on Ph.D. programmes (52 people) was fewer compared to those who enrolled on M.Sc. (94 people). From these Ph.D. students, 36 graduated successfully (data not shown).

The main reason that students do not undertake nematology studies appears to be based on a lack of exposure to this discipline at the undergraduate level (B.Sc.). Simply put, no exposure to nematology as an undergraduate obstructs any further involvement, through a lack of knowledge or awareness of the topic. A FGD held with nematology professors from Kenyan Universities in January 2018 highlighted that the absence of stand-alone courses on nematology at B.Sc. level is a key determinant hampering nematology uptake as a professional discipline. Whenever nematology is taught within tertiary education, it often serves as a complementary component of other curricula (*i.e.*, plant protection, entomology) and mainly at graduate level and is, therefore, rarely a stand-alone course unit. An example of this is in Zimbabwe, where nematology is taught as a topic within Plant Pathology at the Africa University (AU) in the undergraduate programme (AU, 2017), while at Midlands State University and the University of Zimbabwe it is a component of a broader course module at both B.Sc. and M.Sc. level (Z. Sibanda and M. Tavagwisa, pers. comm.). In Malawi, Lilongwe University of Agriculture and Natural Resources (LUANAR) appears to be the only university that teaches nematology in the country but only as a unit within Plant Pathology (D. Kachigamba, pers. comm.). During the last decade, however, the teaching of nematology has begun to change in some African universities, triggered, in some cases, by a policy-enabling academic environment influenced by the appointment of trained nematologists in lecturing positions (see section ‘*A nematological braindrain in Africa*: *is there a future in science?*’). For example, since 2002-2005 the National Universities Commission (NUC, 2017) encouraged specialisation in agriculture across Nigeria, resulting in increased numbers of Crop Protection departments, in which nematology has featured prominently. Under the auspices of these internal policy changes, it is estimated that from the Nigerian faculties of agriculture that teach nematology at the undergraduate level, about 10% of the students that apply for Crop Protection at postgraduate level (M.Sc. and Ph.D.) opt to study nematology (Claudius-Cole & Fawole, 2014). Nonetheless, progress has been slow; at the University of Nigeria Nsukka (Ikoro *et al.*, 2012) between 2001 and 2010, just one student enrolled for a Ph.D. in nematology, representing the lowest level of enrolment (3%) under a curriculum at this university. At Makerere University (MAK) in Uganda, nematology is an elective stand-alone course at M.Sc. level, whilst two courses are taught, along with entomology, at B.Sc. level (H. Talwana, pers. comm.). In Kenya, Kenyatta University (KU), Moi University (MU) and Embu University (UoEm) all have nematology as a stand-alone or shared undergraduate topic; at Jomo Kenyatta University of Agriculture and Technology (JKUAT), Nairobi University and KU, a specialised M.Sc. course unit in plant nematology is offered, while nematology is scheduled to be a course module within an existing M.Sc. programme at MU. Improvements are also gradually being observed in Ethiopia, largely at the M.Sc. level; in 2015, nematology was taught only at Haramaya University (HU) and Hawassa University (HU) (Abebe *et al.*, 2015) but has since been included in the curricula of Jimma University (JU), Ambo University (AU), Mekelle University (MU), Debre Berhan University (DBU), Madda Walabu University (MWU) and Bahir Dar University (BDU). At JU and HU, there are currently 10 M.Sc. and 6 Ph.D. students conducting research in nematology (B. Hailu Meressa and A. Seid Ebrahim, pers. comm.); however, across the spectrum of accredited Ethiopian public universities that offer agriculture and biological sciences just seven of the 30 include nematology in their curricula (Ministry of Education, Federal Democratic Republic of Ethiopia, 2011). Noteworthy, however, is that nematology is seldom taught as a full course at the B.Sc. level anywhere in the world but is mostly taught at the M.Sc. level, often with just a single course.

We also requested interviewees to identify the academic institutions that played a key role in building their capacity in nematology; this was formulated *via* a multiple-choice answer, to capture institutions at B.Sc., M.Sc. and Ph.D. levels. The principal source of nematology training in SSA was unmistakably the International M.Sc. of nematology at UGent (PINC/EUMAINE/IMaNema) (37.6%), followed by African Universities (26.2%), IITA (15.6%), international universities (8.5%), the International Centre for Insect Physiology and Ecology (*icipe*) (5.7%), international research centres (3.5%), and national research programmes in Africa (2.8%). Considering these results, it is safe to say that UGent’s nematology M.Sc. programmes through the scholarship platforms constitute the backbone of nematology capacity building in SSA.

#### A nematological ‘brain-drain’ in Africa: is there a future in science?

Following the independence of various African countries during the 1960s, the emigration of qualified Africans became apparent (Meyer, 2001; El-Kawas, 2004; Labonte *et al.*, 2007; Beine *et al.*, 2008). This migration of highly educated individuals from SSA has persisted, growing by 95% over the past decade, reaching 1.8 million people in 2010/2011. This represented the highest proportion of educated migrants among all non-OECD regions, and 37% of all SSA emigrants (OECD, 2015). Socio-economic factors behind this migration vary, although the lack of professional opportunities for graduates in SSA, as well as immigration policies of reception countries that prioritise educated professionals are key determinants (Beine *et al.*, 2008). In East Africa the main factors attributed to the ‘brain drain’ and the low uptake of nematology in SSA are: *i*) the lack of financial support to conduct research; *ii*) limited infrastructure and equipment; and *iii*) relocation into other roles (*i.e.*, entomology) at their job placement (Abebe *et al.*, 2015; Talwana *et al.*, 2015). There is no information available for the rest of SSA, but it could be speculated that such challenges are similarly applicable across SSA.

#### Where do nematology trainees develop their professional careers?

In our survey, we asked SSA interviewees if they had been able to pursue a scholarly career in nematology after graduation. For those who answered ‘Yes’, the Track A ‘Scholarly/scientific career-oriented path’ was designed; this provided nine questions aimed at characterising their engagement in science and/or academia and to trace the outputs of the same, including scientific papers, student mentoring and funding sources. The ability of the interviewees to develop a professional career in nematology under Track A was higher than envisaged: of 124 respondents, 50.1% (17 women, 43 men, 3 no reply) confirmed a scholarly career/research path, primarily in nematology-related positions in their native countries (69.5%), in other African countries (3.4%) and outside Africa (27.1%). Following this track, research is the main activity developed by interviewees (35.6%; 58 answers) at their current positions, followed by education in nematology at university level (28.2%; 46 answers), administrative work (24.5%; 40 answers) and other (6.1%; 10 answers); the latter mainly relates to extension services and outreach to communities. This was a multiple choice option question that enabled interviewees to provide information on jobs entailing multiple roles.

**Fig. 4:**
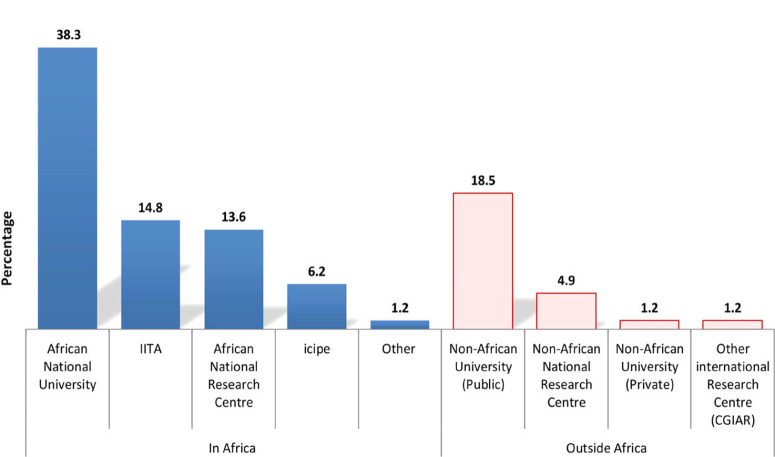
Percentage of responses from sub-Saharan Africa nematologists related to the African and foreign institutions where they developed their professional careers.

We also set up a multiple-choice question to capture an understanding of the type of institution where careers were developed. In this answer, 60 interviewees (74.1%) indicated that their scientific/scholarly careers involved institutions within Africa, 21 respondents (25.9%) also mentioned non-African institutions (Fig. 4), while in some instances, respondents answered positively to both. As career development institutions within the SSA region, respondents listed African universities (38.3%), IITA (14.8%), African national research centres (13.6%), *icipe* (6.2%) and another (1.2%), representing a post at a national Plant Quarantine and Phytosanitary Services workplace. Among the institutions outside SSA, public universities ranked highest (18.5%), followed by national research centres (4.9%), private universities (1.2%) and other (1.2%), which equated to the International Potato Centre (CIP).

**Fig. 5:**
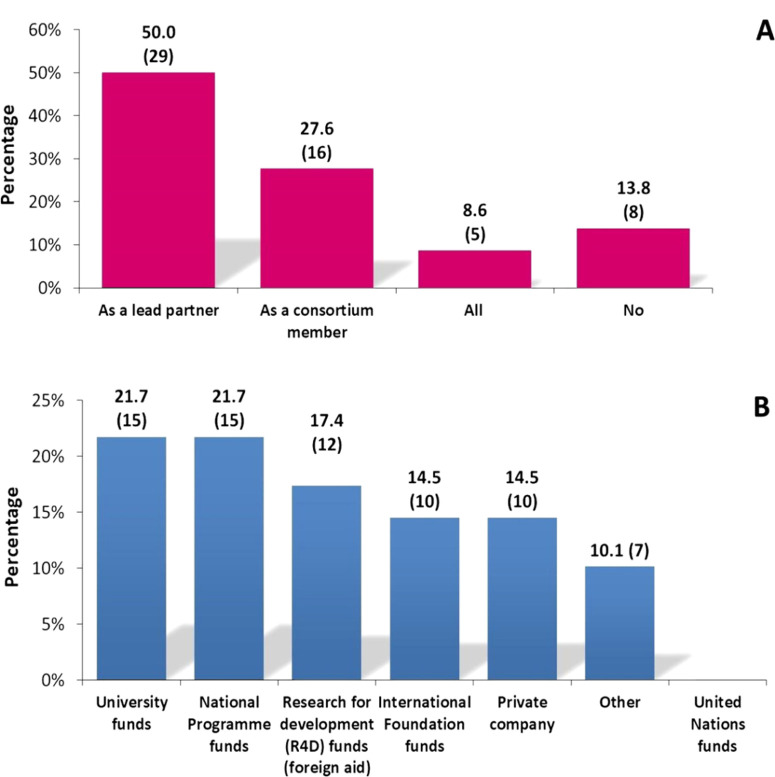
A: Type of engagement by respondents on track A ‘Scholarly/scientific career-oriented path’ (see Table S1 in the online edition of this journal, which can be accessed *via* brill.com/nemy) related to leadership on research activities; B: sources of funding for nematology research acknowledged by interviewees that responded to this track; in parentheses, the number of responses for each category.

Regarding the field of nematology expertise, most respondents are currently active, working with PPN (84.7%), while a few work with entomopathogenic nematodes (EPN) (8.5%), free-living nematodes (1.7%) or taxonomy and systematics (1.7% each); two respondents (3.4%) indicated that they combine research in two of the previous fields, PPN + EPN and PPN + free-living nematodes; no responses were provided for work related to ecology or for nematode physiology. The main drivers for selecting current fields of expertise within nematology were the academic background (69.5%) and the mandate of their institutions (17%), while options such as ‘More funding possibilities, as opposed to other disciplines’ or ‘The existing research means availed to me (*i.e.*, research facilities, infrastructure, and research support)’ were answered by 1.7% and 8.5%, respectively; two were invalid answers. In a multiple choice question, most interviewees indicated that they conduct research either as a lead partner (50%), as a member of a research consortium (27.6%) or as both (8.6%) (Fig. 5A); eight respondents (13.8%) were not conducting any research at the time that this interview was undertaken. National programmes and university funds (21.7%, each with 15 answers) were the main financial sponsors for research, followed by research for development (R4D) aid (foreign funds) (17.4%), international foundation funds and private companies (14.5%, each with ten answers). A number of answers indicated ‘others’ (10.1%), regarding ‘own funds’ (six answers) and government (one answer) for financial sponsoring. There was no acknowledged financial support from any United Nations agencies (Fig. 5B).

The scientific production of scientific nematology papers from SSA for SSA

**Fig. 6:**
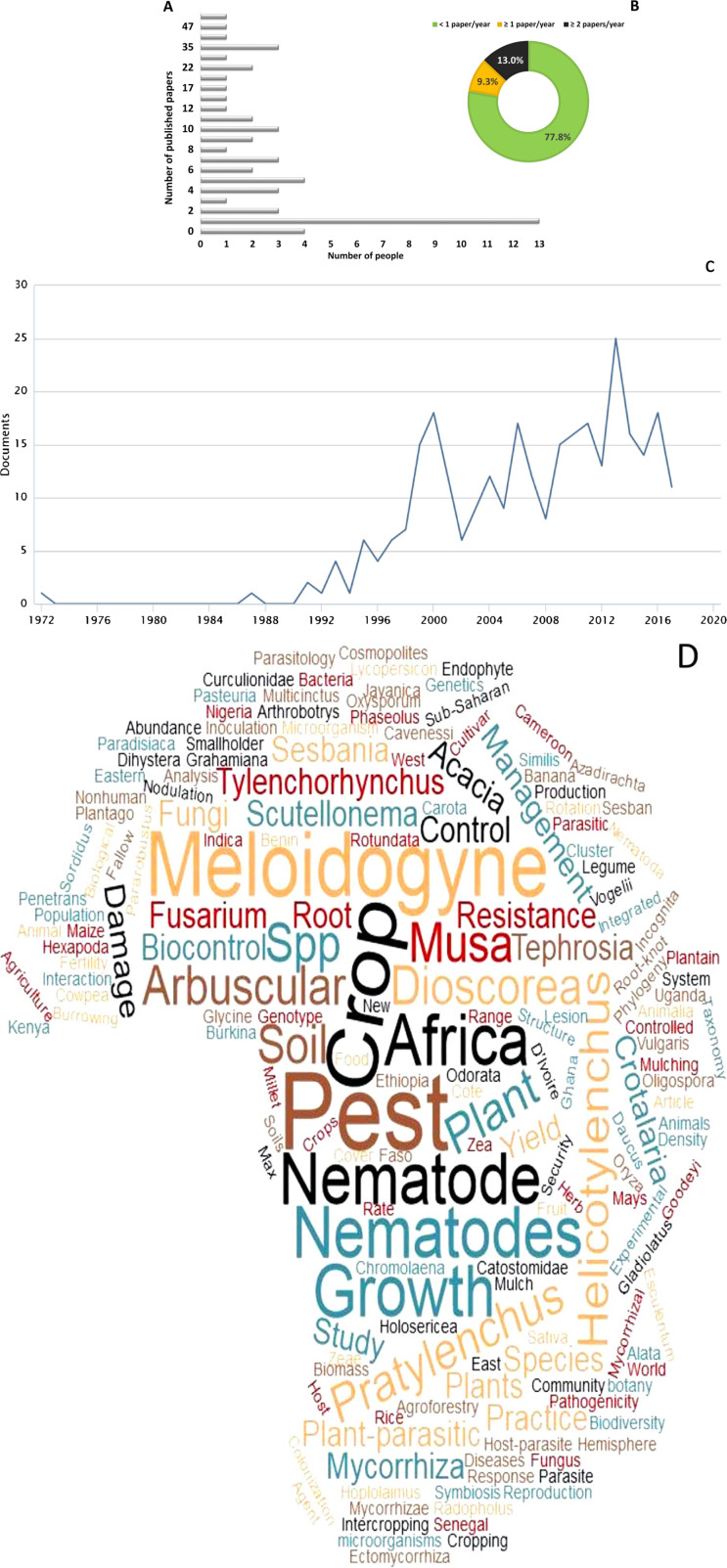
A: Frequency of publication and percentage of publications per year by sub-Saharan Africa interviewees; B: Average number of papers published by interviewees per year; C: Number of publications and reports related to nematology in SSA published in peerreviewed journals available in Scopus since 1972 to 2017; D: WordCloud created with the words obtained from the key words of the abstracts contained in papers retrieved from the Scopus searched.

Within Track A, we also investigated data related to student mentorship and publication of scientific papers. Respondents mentored a healthy number of students (mean ± SE (range)) for their B.Sc. 4.8 ± 10.13 (0-60), M.Sc. 2.4 ± 2.6 (0-11) and Ph.D. 1.5 ± 1.7 (0-5). However, despite these figures, the overall number of publications reported was very low; 54 respondents published an average of 11 papers (10.9 ± 15.81) over the past 15 years, representing 0.7 publications per person per year. The number of publications varied widely among individuals (range 0 to 90) although approximately 80% indicated they had produced more than one paper per year, while the most frequent publication rate was just one in 15 years (Fig. 6A, B). Seven respondents (13%) produced 30 or more papers in 15 years, equivalent to two or more papers per person per year.

A filtered search on the Scopus Database (‘plant parasitic nematodes’ w/africa; see Table S3 in the online edition of this journal, which can be accessed *via* brill.com/nemy) related to the study of PPN that affect food security within Africa, showed 296 records for peerreviewed research papers and reviews since 1972. This is a small proportion considering the global index for publications in our study area (Ciancio, 2015). However, although the overall number of publications in nematology from SSA is low, particularly before 1990 (Fig. 6C), there has been a steady progressive increase over time, from one paper per year in 1972 to 11 papers per year in 2017. In line with the data from our interviews, the search on Scopus also shows that a high number of publications originate from just a small number of institutions and individual nematologists, and that this research is primarily related to nematode species impacting negatively on food security in SSA and management options (Fig. 6D). Most Thomson-indexed papers, however, have been published in specialised nematology journals, such as *Nematology* (31 papers) or *Nematropica* (18). Other outlets include local journals that have limited international visibility or accessibility, are in local language or are in non-Thomson indexed journals, although it is difficult to quantify the number of nematology publications scattered under this broad category.

An aim of nematology researchers in SSA should be to increase scientific publication in internationally respected, peer-reviewed journals. This would increase their visibility and facilitate national and international nematology networks across SSA and would impact on how local researchers access human capital and financial resources from donors outside the continent. Greater access to publishing in open access (OA) is also a barrier, but other obstacles that need to be overcome to increase publication rates include a lack of supporting policies, availability of online repositories and databases, or adequate training of librarians on ICT technologies (Chisenga, 2000; Odero & Mutula, 2007; Fox & Hanlon, 2015).

To encourage publication of research results, some universities in SSA have initiated policies that require postgraduate students to publish at least one paper in order to qualify for graduation at M.Sc. or Ph.D. level (*i.e.*, KU and JKUAT in Kenya). However, while this policy is advantageous to the student, to the institution and to the discipline, this pressure to publish can have potential negative consequences. Over the past decade there has been an explosion in the number of predatory publishers and journals that lack a robust and transparent peer-reviewed process, leading to sub-standard scientific quality. These journals have become popular outlets for otherwise unpublishable work, at a cost both financial and to the reputation of the researcher. While some authors (11%) in wealthier countries will personally pay article processing charges (APCs), more (39%) are willing to do so in less-developed countries (Solomon & Björk, 2011), helping to explain why predatory journals that generally have lower APCs (US$ 178) have become popular in these regions (Shen & Björk, 2015). In Nigeria, the country in SSA with the highest number of predatory publishers and journals, the negative impact for scientists publishing in these journals is demonstrated through the 1580% ratio of papers published in predatory journals *vs* Web of Science-indexed journals in 2013-2014, the highest in the world (Shen & Björk, 2015; Ayeni & Adetoro, 2017). It is important, therefore, to steer our SSA nematologists along a more positively impacting pathway, through better awareness of publishing ethos and principles. Mechanisms to reduce or waive APCs in Web of Science indexed journals for researchers in poorresource economies could therefore be highly beneficial for SSA. For instance, The Bill and Melinda Gates Foundation has commendably initiated a policy to cover the costs of OA for all publications arising from the projects that they support.

#### What does nematology look like in a non-research career-oriented path?

Track B was designed to get a better understanding of the main limiting factors in SSA for nematologists (with M.Sc. or Ph.D.) to pursue a career in nematology in SSA. Five questions aimed to establish the reasons why a nematology career was not followed or how their nematology background benefitted their current job. Contrary to the commonly held assumption that postgraduates do not return to Africa for work, most respondents are conducting their careers in SSA, with 80% in their own countries, 8.6% in other African countries and just 11.4% outside Africa.

**Fig. 7:**
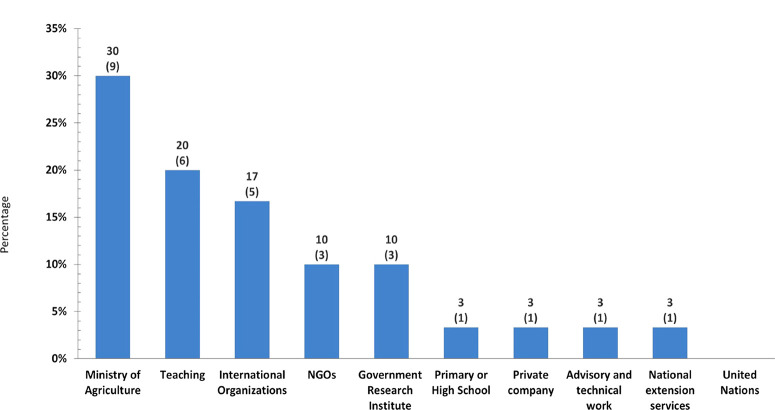
Organisations where sub-Saharan Africa nematologists are conducting their professional careers non-related to nematology as a research/scientific discipline; in brackets, the number of responses for each category.

The lack of grants for nematology at the Ph.D. or postdoctoral level was the most frequent response (44.4%) for not pursuing nematology, followed by the lack of stable scholarly/scientific positions (33.3%), in line with the challenges that have previously been reported in the literature (Abebe *et al.*, 2015; Talwana *et al.*, 2015). The options of ‘Willing to redirect careers to other disciplines/activities’ and ‘Personal circumstances’ wereminor reasons. Interestingly, no respondents had any wishful intention to move away from nematology. Track B was also intended to determine if graduate nematologists that could not pursue a scholarly/research track are (or have been) applying their nematology expertise in their professional careers, while outside of academia. Approximately two-thirds of the respondents (67%) indicated that nematology is somehow applied in their current positions, while for a third it is not. Most respondents are involved in generalist agricultural advisory and technical work (44.2%) – not strictly linked to nematology – with the Ministry of Agriculture (MoA) in their own countries or teaching subjects unrelated to our discipline in African universities (29.4%), with just a few employed in national extension services and agricultural policy development (Fig. 7). Of 34 respondents, five gave invalid answers as they were involved in nematology-related Ph.D.s.

#### Personal perceptions

**Fig. 8:**
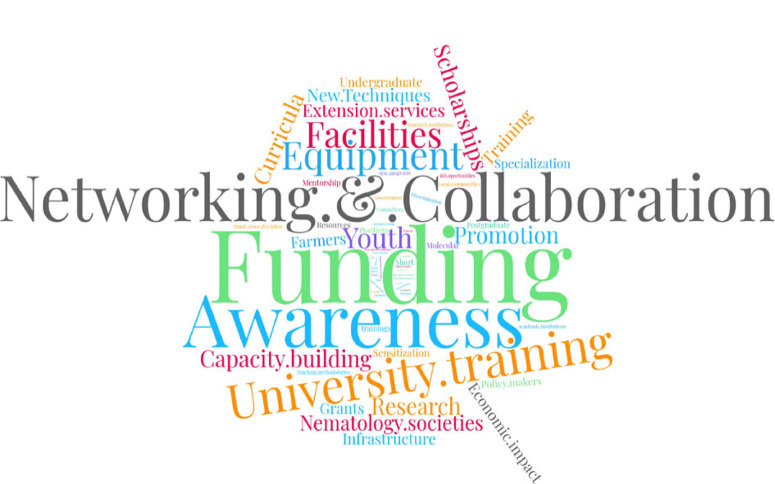
Word-cloud of respondents’ perceptions on what is required to promote nematology in sub-Saharan Africa.

In our survey, African nematologists were requested to identify areas for the improvement of nematology as a research discipline in SSA, which was developed into a word-cloud (Fig. 8). A high proportion of respondents indicated the need for nematology to be included in the curricula at B.Sc. level as a stand-alone discipline in African universities. Terms were also mentioned that indicated the need to integrate young professionals better. Beyond this, there was emphasis on funding, access to equipment and infrastructure (Fig. 8).

**Fig. 9:**
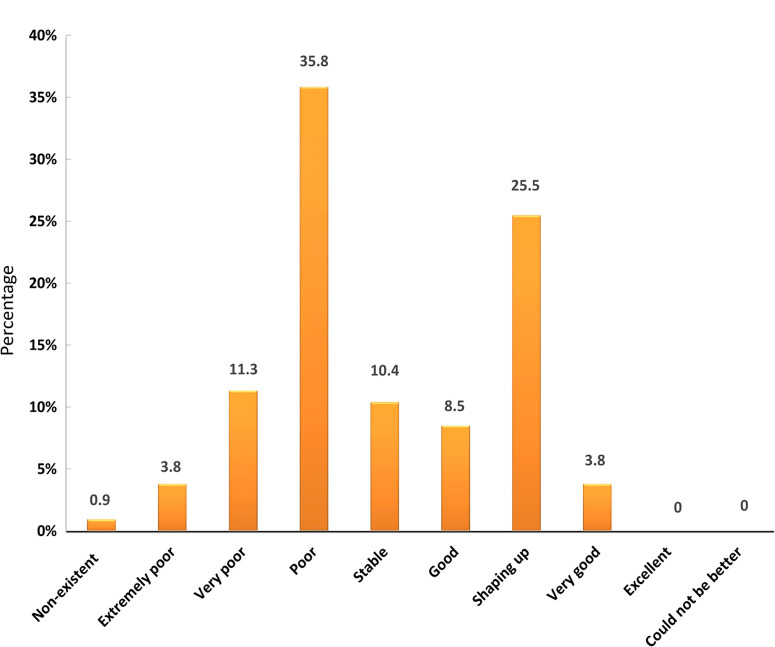
Ranking of the level of personal satisfaction of interviewees related to the current status of nematology in sub-Saharan Africa.

Surprisingly, networking featured prominently towards the promotion of nematology both within and outside SSA. Some respondents proposed tapping into the African diaspora to establish and consolidate global networks of collaboration between Africa and the EU, North America or Asia. Refresher courses or the updating of technologies/methods (*i.e.*, molecular techniques) through short courses/workshops at the university level for students and professionals are needed to enhance nematology awareness. Respondents from SSA also urged themselves to address the weak coordination of the few regional nematology networks in Africa and gain more visibility both locally and internationally. Finally, when asked to gauge the general health status of nematology as a discipline in SSA, most felt that the situation is non-existent to poor (51.8%); a stable situation was indicated by 10.4% of interviewees and, on a more positive note, 37.8% of the respondents said it is good to very good (Fig. 9).

### From Knowledge To Action

#### Nematology at the grassroots level in African agricultural systems

During its implementation, the NIESA project was successful in its promotion of nematology as a science/research field in SSA but it only tangentially addressed policy transformation and increased farmers’ awareness (Waceke *et al.*, 2012) due to its project-based nature and the limited funding period. As seen in the previous section, our study shows that despite the increasing number of nematology professionals over the last two decades, this has yet to translate meaningfully into better knowledge, understanding and advice on nematology within extension and national advisory services in SSA. Numbers are increasing, as indicated under the filtered responses under the option ‘Advisory and technical work’ from Track B; it undoubtedly takes a lengthy period of time to transcend and impact across the system as a whole (Fig. 4).

This situation, therefore, provides cause for concern if we consider that researchers have identified smallholder farmers’ access to extension services as a key factor to increasing agricultural productivity (Lee, 2017). Compounding the situation, pests and diseases are identified as among the greatest threats to crop productivity in SSA (Savary *et al.*, 2012; Vanlauwe *et al.*, 2014), and root-knot nematodes as the single greatest biotic threat (Coyne *et al.*, 2018). Farmers’ access to good quality information and advice is thus critical to reversing the current trend in agricultural production in SSA. A survey in Malawi, Swaziland, Zambia and Zimbabwe highlights the paucity, or absence even, of nematology expertise in national research and agricultural extension services (Sakala & Soko, 2011). The situation is reflected across many countries but when present, nematology expertise is often overstretched to cover a large mandate. In Cameroon, the Centre Africain de Recherche sur Bananiers et Plantains (CARBAP; http://www.carbapafrica.org/index.php/2013-09-13-16-08-01/expertises) has two nematologists (1 Ph.D. and 1 M.Sc.) who cover banana and plantain research in Cameroon, Chad, Central African Republic, Democratic Republic of Congo, Congo, Gabon and Equatorial Guinea. In addition, the nematologists at CARBAP provide nematology training and advice to students and farmers across Cameroon (Franklin Lombi Mbongo, pers. comm.). In some instances, particularly in Eastern Africa, nematology expertise is healthier, albeit remaining low (Talwana *et al.*, 2015). In other situations, the necessary facilities and equipment to conduct basic nematology are available but lack nematologist expertise, such as in Somalia (Dario Cipolla, FAO Somalia, pers. comm.).

Beyond the lack of technically qualified human power, SSA extension programmes also suffer from poor monitoring on the quality of the services provided, absence of conducive agricultural policies for stimulation of agricultural innovation and technology transfer, and limited understanding on the uptake of such agricultural innovations (Davis, 2008; Ragasa *et al.*, 2016; Sartas *et al.*, 2018). By tailoring farmers’ needs through a demanddriven approach (‘bottom-up’) that uses participatory research models within the socio-economic and cultural context of the rural communities, extension services can be improved (Davis, 2008). However, this approach would likely overlook the inclusion of nematology given the limited understanding and perceptions of smallholder farmers in relation to agricultural problems caused by nematodes (Speijer *et al.*, 2001; Osei *et al.*, 2004; Kagoda *et al.*, 2010; Wairegi *et al.*, 2010; Mulumba *et al.*, 2012), in addition to these being regularly overlooked or ignored by crop protectionists when assessing community needs (Abang *et al.*, 2014; Mengistie *et al.*, 2017). In those situations where farmers associate PPN with production losses, plant health problems persist due to: *i*) limited access to quality extension services; *ii*) inadequate training and access to nematode management measures (including nematicides); and/or *iii*) hampered access to agricultural innovations (DeWaele & Elsen, 2007). For example, in Western Kenya, communities that were frequently visited by rural agents were more aware of PPN damage and symptoms than communities that did not receive technical support. Despite this, farmers from both communities experienced similar damage and yield losses from PPN due to inadequate information and inefficient measures provided to them to manage PPN (Nchore Bonuke, 2016). In Egypt (and the Middle East), however, where university training in nematology is seen as more ‘nematodeinclusive’ than in SSA, overarching structural challenges similarly hamper progress in addressing PPN damage (Abd-Elgawad, 2014). Improving farmer awareness of nematology at the grassroots level requires national advisory services to be informed and perceptive of PPN issues. This can only be achieved through the provision of adequate educational and agricultural policy support, considering also an inclusive approach to gender mainstreaming. Access to one additional year of education has been shown to increase women’s salaries by 20% (Psacharopoulos & Patrinos, 2002), whilst it is estimated that it costs more than US $1 billion per annum to countries that fail to provide equal education to boys and girls (GPE, 2015). Female extension service providers, mentors and/or lead farmers make a differential impact on the food security of their communities, as often their female peer farmers have a better chance to access knowledge transfer if this is provided by women (Bello-Bravo *et al.*, 2011; Mudege *et al.*, 2016; Achandi *et al.*, 2018); their presence can be a transforming factor for improving yields of smallholders in SSA (Achandi *et al.*, 2018) where 40% of the human power is women (Palacios-López *et al.*, 2017).

#### Future initiatives for the support of nematology in sub-Saharan Africa

There are currently few initiatives that directly target nematology within SSA. Some broader, all-encompassing initiatives take nematology into account under the banner of crop protection or plant health, which strongly benefit a more holistic understanding of the subject. Initiatives such as PEARL create a highly supportive and facilitatory environment, enabling young researchers to carve out a niche for nematology within their national research system. NIESA similarly supported the creation of an image of nematology within national programmes as an important discipline upon which a career could be built. In 2017, the IMaNema M.Sc. programme gained funding for an additional 5 years, including scholarships to students from SSA and further afield, extending the programme to include *in situ* training modules in collaboration with IITA and *icipe* (Kenya) and Jimma University (Ethiopia). IMaNema is, to our knowledge, the only nematologyspecific funded initiative for capacity building currently active in SSA.

#### How new technologies can help nematologists in sub-Saharan Africa to learn more and stay connected

To promote nematology effectively in Africa, digital technologies and learning platforms (DLP) will be vitally important. The availability and quality of internet access in SSA has greatly improved over the last decade or so, from 17 million access points in 2005 to an estimated 172 million in 2014. In countries with stable internet networks (*i.e.*, Ghana, Kenya, Tanzania and Zambia), 68.3% of users consider it useful for access to agricultural information on crop and livestock management (Guerriero, 2015). To optimise access to and awareness of nematology, IMaNema is developing a nematology digital learning platform (NDLP) (source: http://www.pinc.ugent.be/index.asp?p=2509&a=2509) to support students, lecturers, researchers, agricultural extension workers and nematology professionals globally, with free online tutorials and training materials. This digital tool is intended to have a significant impact in environments such as encountered in SSA, where financial constraints regularly impede both student and researcher access to educational resources. A secondary objective for this NDLP is to create a point of contact and networking for SSA nematologists, to share research results, material, training opportunities, or simply to interact with each other. Although various nematology society platforms and project-related websites already exist, a common, centralised, popular nematology NDLP could serve as a focal point. The Nemaplex portal (source: http://nemaplex.ucdavis.edu/Uppermnus/topmnu.htm) is a remarkable on-line repository that has a wide range of training contents in English and Spanish, pictures, tools (*i.e.*, NINJA) and learning resources, which has been conceived for teaching and training nematology but not as a ‘meeting point’ for professionals around the world.

Information and Communication Technologies (ICT) such as radio and telephone are also effective communication channels for the delivery of agricultural knowledge and technical guidance across Africa (Asenso-Okyere & Mekonnen, 2012), especially considering that in SSA the vast majority of people have access to radio and mobile networks (EU, 2017) in a scenario where the ratio of farmers to extension officers is extremely high (*i.e.*, 753:1 in Kenya, according to Sanga *et al.*, 2013). Such communication channels are especially relevant for female farmers, who have less access to training opportunities outside their households or who are otherwise prevented from accessing extension services due to cultural barriers (Quisumbing *et al.*, 2014; Akwii *et al.*, 2017; Hudson *et al.*, 2017). To ensure that ICT training is effective, content should be gender-and ethnicity-sensitive and take into consideration literacy, language and learning constraints (Mudege *et al.*, 2016).

#### Nematology networks and societies

There are 16 registered nematology societies under the International Federation of Nematology Societies (IFNS) (http://www.ifns.org/home/index.php/membership/). In Africa, Northern African (NA) countries (definition of NA and SSA countries, UN: https://unstats.un.org/unsd/methodology/m49/) have been quite active through the Afro-Asian Society of Nematologists (started 1990) and the Egyptian Society of Agricultural Nematology (1993). Currently in SSA there are two societies: the Nematological Society of Southern Africa (NSSA) and the Nigerian International Society of Nematologists (NISON). NSSA (1972) is an active society with bi-annual meetings, a website and the production of several newsletters. It also serves *de facto* as the African society, which serves to keep the nematology community connected. In South Africa, nematology is also included in the undergraduate curricula at several universities, as well as in the public (extension and research) and private sectors. NISOM was created in 2011 and currently has more than 50 active members that meet biannually. It also edits the peerreviewed journal *Nigerian Journal of Nematology* (NIJON).

To date, no Pan-African nematological society has been established, although this was highlighted as a major milestone towards strengthening and uniting nematology expertise at the SSA regional level by our interviewees. Efforts towards building a regional network in SSA were undertaken during 2011 (FANRPAN, 2011) and 2013 (PAEPARD, 2013) through the NIESA initiative. However, establishing and maintaining a society and an active network requires energy, willing participants and funding to sustain activity and interest, resources that are not always readily available.

## Conclusion

Training in nematology is limited in comparison to many other plant health disciplines and continues to suffer numerous challenges. However, through the data collected during our study, the prospects for the future of nematology in SSA appear bright, contrary to traditional belief by African and non-African nematologists. The data show that more students from SSA have received training in UGent programmes than any other regions, and that most students return home and find a qualified job on completion of the course. Despite this, a lack of nematologyspecialised posts frustrate the situation, although an increasing awareness and support for nematology is seeing its increasing inclusion as a stand-alone topic in undergraduate courses in SSA. Through greater exposure to nematology, undergraduates are increasingly becoming aware of the subject as a real topic, an interesting and important discipline, and a potential future career as nematologists. Gradually, the increased recognition of nematology at undergraduate level and in the plant health and crop protection arena in SSA will ultimately lead to the generation of jobs for nematologists. There is no denying the long road ahead, although it is a road that appears far more appealing and positive than it did previously. Hence, regardless of whether our interviewees found a position in academia or out of it, the skills and knowledge they gained have allowed them to position themselves in the local job market and to bring back to Africa their know-how.

Despite the fact that data from the UGent M.Sc. programmes cannot be considered the only source to evaluate capacity building on nematology in SSA, the information regarding gender, origin and graduation rate of this vast database can be used as an excellent proxy to investigate and quantify the impact of these programmes in the promotion of nematology in SSA over the last 25 years. Our analyses show that the UGent nematology curriculum has been one of the most critical driving forces for training African students. It is important to mention that, despite the core of the nematology training at postgraduate level for SSA students seemingly taking place at the M.Sc. level, those who are able to pursue a Ph.D. have mainly completed their degrees with scholarships and financial support obtained through universities outside Africa, mainly in the EU, South Africa and in the USA, and hence the role of such non-African universities on promoting higher education levels for nematology has to be acknowledged (R.A. Sikora, D. De Waele, A.P. Malan and H. Fourie, pers. comm.).

The take-up of nematology by African universities is also contributing significantly, along with the mentorship and financial support through specific projects and international institutions (*i.e.*, NIESA, *icipe* and IITA). However, the inclusion of nematology at undergraduate level in SSA remains weak and could be attributed to weak university policies, and not necessarily to a lack of sufficiently trained staff, as has similarly been observed elsewhere (Moreira Souza, 2017). To address food security constraints in SSA, nematology will need to be taken seriously and included as a key issue, alongside other plant health disciplines; projections indicate that nematology will gain more relevance in the next 50-100 years in tropical agriculture as human populations increase in a climate change context that is likely to jeopardise food security for future generations (Sikora *et al.*, 2018). The data gathered in our research have also demonstrated that while trained nematologists are mostly working at the academic level, the information flow to the community and smallholder farmers remains insufficient. In particular, more SSA women should be brought on board, not only to pursue both Tracks A and B, but especially to gain a critical mass of qualified women conducting nematology awareness and advisory support to rural communities and to reach more female smallholder farmers in Africa.

## Recommendations to promote nematology in SSA

- Support SSA countries to develop educational policies to include nematology as a discipline in the university curricula in Africa at B.Sc. level (for Agriculture and Biology), to generate graduates ready to provide continuous technical capacity to the national agricultural programmes, extension services, NGOs and private companies. The inclusion of nematology courses that are more soil-ecosystem relevant could help to highlight the need for creating capacity in this discipline, beyond the plant health thematic area.
- Provide continued support to the IMaNema programme in its efforts to provide training in a framework of strategic partnerships with countries in SSA and elsewhere, allowing for a closer collaboration between partners and the involvement of students and alumni in research and educational initiatives. As much as possible, funding sources of such training programmes should be diversified and secured in the medium to long-term to guarantee their continuity.
- Create long-term specific projects/programmes dedicated to the support of extension services from national organisations, non-governmental organisations and private companies in SSA that should be promoted and coordinated at the Ministry level.
- Reinforce networking of nematologists across the SSA region, through re-activation of the former NIESA website and online platforms, such as communities of practice (CoP); possibly establish a society of Association of African Nematologists that could agglutinate nematologists and effectively link with other international nematological societies.
- To ensure future generations of nematologists in SSA, more applicants from non-anglophone SSA countries are needed. More inclusion of such participants by organising training and promoting nematology in languages other than in English. Providing scholarships for nationals not on the VLIR-UOS country list.
- Continuous efforts to include women, get more data on the career path of women after graduating from the M.Sc. nematology programme at UGent and build a support strategy based on results. Access to alternative scholarships, short trainings, or the use of social media and digital learning platforms/tools, should be explored too in order to reach out to local communities and universities.
- Provide improved support to publish in peer-review international journals in OA and populate online repository databases and contribute to transform all the nematology grey literature and make it accessible to the public.
- *viii*) Focus and expand existing digital initiatives. Active collaboration and contributions on one platform are important.

## Figures and Tables

**Supplementary Table S1:** SurveyMonkey questionnaire and Key Informant Interviews.

1.-County of origin (only Africa): (Only single option is valid)	•Angola
•Benin
•Botswana
•Burkina Faso
•Burundi
•Cameroon
•Cape Verde
•Central African Republic
•Chad
•Comoros
•Congo (Brazzaville)
•Congo (Democratic Republic)
•Côte d’Ivoire
•Djibouti
•Equatorial Guinea
•Eritrea
•Ethiopia • Gabon
•The Gambia
•Ghana
•Guinea
•Guinea-Bissau
•Kenya
•Lesotho
•Liberia
•Madagascar
•Malawi
•Mali
•Mauritania
•Mauritius
•Mozambique
•Namibia
•Niger
•Nigeria
•Réunion
•Rwanda
•Sao Tome and Principe
•Senegal
•Seychelles
•Sierra Leone
•Somalia
•South Africa
•Sudan
•Swaziland
•Tanzania
•Togo
•Uganda
•Western Sahara
•Zambia
•Zimbabwe
2.-Gender: (Only single option is valid)
•Female
•Men
•I prefer not to disclose my gender
3.-Age: (Only single option is valid)
•Below 25
•From 25 to 35
•From 36 to 45
•Above 46
4.-Please, define your highest academic qualifications as of today (Only single option is valid)
•B.Sc.
•Msc
•Ph.D.
•Other (Please, indicate):
5.-Did you ever receive specific training on nematology at any moment while pursuing your university studies, either inside or outside Africa? (Multiple options are valid)
•At B.Sc. level
•At M.Sc. level
•At Ph.D. level
•No, I never received training on nematology during my studies.
6.-If Yes to Question #5, where did you receive the training in nematology? (Multiple options are valid)
•National University in Africa
•International University
•International Master of Science in Agro-and Environmental Nematology (Ghent University)
•National Research Centre in Africa
•IITA
•icipe
•CIMMYT
•ICARDA
•Other International Research Centres
•Other (Please, indicate):
7-Did you conduct specific research on nematology as part of your B.Sc., M.Sc. and/or Ph.D. thesis? (Multiple options are valid)
•B.Sc. Yes
•B.Sc. No
•M.Sc. Yes
•M.Sc. No
•Ph.D. Yes
•Ph.D. No
8-Did you graduate successfully from your nematology work during your M.Sc. and/or Ph.D.? (Multiple options are valid)
•M.Sc. Yes
•M.Sc. No
•Ph.D. Yes
•Ph.D. No
9-Have you been able to pursue a professional scholarly/research career in nematology after your graduation? (Only single option is valid)
•Yes
•No
10-If Yes to Question #9, where did you develop your career in nematology? (Only single option is valid)
•In my own country
•In other African countries
•Outside Africa
11-If Yes to Question #9, Please indicate what type of institution? (Multiple options are valid)
•African National University.
•Non-African University (Public)
•Non-African University (Private)
•African National Research Centre
•Non-African National Research Centre
•IITA
•Other international Research Centre (CGIAR)
•icipe
•Other (Please, indicate):;
12-If Yes to Question #9, What type of activities do you conduct in your current position (please provide approximate time %) (Multiple options are valid up to a maximum value of among the four questions 100%)
•Research work: %
•Teaching work: %
•Administrative work: %
•Other (Please, indicate):
13-If Yes to Question #9, What is your principal line/discipline of research on nematology? (Only single option is valid)
•Plant parasitic nematodes
•Entomopathogenic nematodes
•Free living nematodes (soil and aquatic)
•Taxonomy and systematics
•Ecology & diversity
•Physiology
•Other (Please, indicate):
14-If Yes to Question #9; What are the main drivers of your current research in nematology? (Only single option is valid)
•My academic background.
•The mandate of my institution/organization.
•The opportunity to access more funding possibilities, as opposed to other disciplines.
•The existing research means availed to me (i.e., research facilities, infrastructure, and research sup-port).
•Other (Please, indicate):
15-If Yes to Question #9; How many B.Sc., M.Sc. and/or Ph.D. students do you have under your supervision? (Open numerical question: each category can go from 0 to 1000)
•B.Sc.:
•M.Sc.
•Ph.D.
16-If Yes to Question #9, have you published papers in the area of nematology in the last 15 years? (For YES option is an open numerical question: each category can go from 0 to 1000)
•Yes; please indicate how many:
•No
17-If Yes to Question #9, Are you currently engaged and conducting activities on any research project on nematology? (Multiple options are valid)
•Yes; as a lead partner.
•Yes; as a consortium member.
•No.
18-If yes to question #17; Where are the funds coming from? (Multiple options are valid)
•National Program funds
•International Foundation funds
•University funds
•United Nations funds
•Research for development (R4D) funds (foreign aid)
•Private company
•Other (Please, indicate):
19-If No to Question #9; If you have not pursued a professional scholarly/research career in nematology after your graduation, why was that?
•No scholarships available to continue in nema-tology research.
•No stable positions available to further pursue nematology research.
•Willing to redirect career to other disci-plines/activities.
•No further interest on nematology research.
•Personal circumstances
•Other (Please, indicate):
20-If No to Question #9; Have you been able to pursue a professional career (non-scholarly) that keeps you engaged (directly or indirectly) in any activities that require and value your academic background on nematology? (Only single option is valid)
•Yes
•No
21-If Yes to Question #20; What professional activities are you currently conducting? (Only single option is valid)
•Teaching
•Advisory and technical work
•Agriculture policy development
•National Extension services
•Sales
•Management
•Other (Please, indicate):
22-If Yes to Question #20; In what country are you currently developing your professional activities? (Only single option is valid)
•In my own country.
•In other African countries.
•Outside Africa.
23-If Yes to Question #20; In what organization are you currently developing your professional activities? (Only single option is valid)
•National extension services
•Ministry of Agriculture
•Primary or High School
•Private company
•United Nations
•NGOs
•International Organizations.
•Other (Please, indicate):
24-Under your own point of view, what is in general the health status of nematology discipline in Sub Saharan Africa? Rate from 1 to 10, where 10 is the healthiest and 1 the poorest. (Only single option is valid)
•1.-Non-existent
•2.-Extremely Poor
•3.-Very Poor
•4.-Poor
•5.-Stable
•6.-Good
•7.-Shaping up
•8.-Very Good
•9.-Excellent
•10-Could not be better
25-And just to finalize: Why do you think nematology is important and what do you think would be needed to promote more research on nematology in SSA? Please explain in detail (Open question)

**Supplementary Table S2:** Search code for Scopus.

( “plant parasitic nematodes” w/africa ) AND ( LIMIT-TO ( AFFILCOUNTRY, “Uganda” ) OR LIMIT-TO ( AFFILCOUNTRY, “Nigeria ” ) OR LIMITTO ( AFFILCOUNTRY, “Kenya ” ) OR LIMIT-TO ( AFFILCOUNTRY, “Senegal” ) OR LIMIT-TO ( AFFILCOUNTRY, “Cameroon ” ) OR LIMITTO ( AFFILCOUNTRY, “Burkina Faso ” ) OR LIMIT-TO ( AFFILCOUNTRY, “Benin” ) OR LIMIT-TO ( AFFILCOUNTRY, “Ghana ” ) OR LIMITTO ( AFFILCOUNTRY, “Cote d’Ivoire ” ) OR LIMIT-TO ( AFFILCOUNTRY, “Ethiopia” ) OR LIMIT-TO ( AFFILCOUNTRY, “Rwanda ” ) OR LIMITTO ( AFFILCOUNTRY, “Niger ” ) OR LIMIT-TO ( AFFILCOUNTRY, “Sudan” ) OR LIMIT-TO ( AFFILCOUNTRY, “Zimbabwe ” ) OR LIMITTO ( AFFILCOUNTRY, “Burundi ” ) OR LIMIT-TO ( AFFILCOUNTRY, “Democratic Republic Congo ” ) OR LIMIT-TO ( AFFILCOUNTRY, “Mozambique ” ) OR LIMITTO ( AFFILCOUNTRY, “Congo ” ) OR LIMIT-TO ( AFFILCOUNTRY, “Madagascar” ) OR LIMIT-TO ( AFFILCOUNTRY, “Seychelles ” ) OR LIMITTO ( AFFILCOUNTRY, “Togo ” ) OR LIMIT-TO ( AFFILCOUNTRY, “Undefined” ) ) AND ( LIMIT-TO ( SUBJAREA, “AGRI ” ) OR LIMIT-TO ( SUBJAREA, “ENVI” ) OR LIMIT-TO ( SUBJAREA, “BIOC ” ) OR LIMIT-TO ( SUBJAREA, “EART” ) OR LIMIT-TO ( SUBJAREA, “MULT ” ) ) AND ( LIMIT-TO ( DOCTYPE, “ar” ) OR LIMIT-TO ( DOCTYPE, “re ” ) ) AND ( LIMITTO ( PUBYEAR, 2017 ) OR LIMIT-TO ( PUBYEAR, 2016 ) OR LIMITTO ( PUBYEAR, 2015 ) OR LIMIT-TO ( PUBYEAR, 2014 ) OR LIMITTO ( PUBYEAR, 2013 ) OR LIMIT-TO ( PUBYEAR, 2012 ) OR LIMITTO ( PUBYEAR, 2011 ) OR LIMIT-TO ( PUBYEAR, 2010 ) OR LIMITTO ( PUBYEAR, 2009 ) OR LIMIT-TO ( PUBYEAR, 2008 ) OR LIMITTO ( PUBYEAR, 2007 ) OR LIMIT-TO ( PUBYEAR, 2006 ) OR LIMITTO ( PUBYEAR, 2005 ) OR LIMIT-TO ( PUBYEAR, 2004 ) OR LIMITTO ( PUBYEAR, 2003 ) OR LIMIT-TO ( PUBYEAR, 2002 ) OR LIMITTO ( PUBYEAR, 2001 ) OR LIMIT-TO ( PUBYEAR, 2000 ) OR LIMITTO ( PUBYEAR, 1999 ) OR LIMIT-TO ( PUBYEAR, 1998 ) OR LIMITTO ( PUBYEAR, 1997 ) OR LIMIT-TO ( PUBYEAR, 1996 ) OR LIMITTO ( PUBYEAR, 1995 ) OR LIMIT-TO ( PUBYEAR, 1994 ) OR LIMITTO ( PUBYEAR, 1993 ) OR LIMIT-TO ( PUBYEAR, 1992 ) OR LIMITTO ( PUBYEAR, 1991 ) OR LIMIT-TO ( PUBYEAR, 1990 ) OR LIMITTO ( PUBYEAR, 1988 ) OR LIMIT-TO ( PUBYEAR, 1987 ) OR LIMITTO ( PUBYEAR, 1972 ) )

Only SSA countries were included with the exclusion of South Africa.

**Supplementary Table S3:** Word Cloud words’ list.

Academic institutions: 2
African Union: 1
Agricultural policies: 1
Awareness: 29
Capacity building: 9
Committees: 2
Conferences: 1
Crop-specific challenges: 1
Curricula: 9
Diagnostics training: 1
Dissemination: 2
Donors: 1
Economic impact: 6
Equipment: 15
Extension services: 7
Facilities: 14
Farmers: 7
Funding: 40
Ghent University: 1
Government: 2
Grants: 6
Icipe: 1
IITA: 1
Infrastructure: 6
International: 1
Job opportunities: 2
Lecturers: 1
Local communities: 1
Mentorship: 3
Molecular: 3
M.Sc. programs: 2
Nematology societies: 8
Networking & Collaboration: 25
New Techniques: 7
Personnel: 1
Policy makers: 4
Positions: 3
Postgraduate: 3
Private: 1
Promotion: 9
Public: 1
Recognition: 1
Regional: 1
Research: 9
Research institutions: 2
Resources: 3
Scholarships: 9
Sensitization: 4
Short trainings: 3
Specialization: 5
Staff retention: 1
Stand-alone discipline: 2
Academic institutions: 2
African Union: 1
Agricultural policies: 1
Awareness: 29
Capacity building: 9
Committees: 2
Conferences: 1
Crop-specific challenges: 1
Curricula: 9
Diagnostics training: 1
Dissemination: 2
Donors: 1
Economic impact: 6
Equipment: 15
Extension services: 7
Facilities: 14
Farmers: 7
Funding: 40
Ghent University: 1
Government: 2
Grants: 6
Icipe: 1
IITA: 1
Infrastructure: 6
International: 1
Job opportunities: 2
Lecturers: 1
Local communities: 1
Mentorship: 3
Molecular: 3
M.Sc. programs: 2
Nematology societies: 8
Networking & Collaboration: 25
New Techniques: 7
Personnel: 1
Policy makers: 4
Positions: 3
Postgraduate: 3
Private: 1
Promotion: 9
Public: 1
Recognition: 1
Regional: 1
Research: 9
Research institutions: 2
Resources: 3
Scholarships: 9
Sensitization: 4
Short trainings: 3
Specialization: 5
Staff retention: 1
Stand-alone discipline: 2
Students: 1
Survey: 1
Taxonomy: 1
Teaching methodologies: 2
Technical research: 1
Training: 7
Undergraduate: 5
University of Florida: 1
University training: 19
Youth: 11
Regional: 1
Research: 9
Research institutions: 2
Resources: 3
Scholarships: 9
Sensitization: 4
Short trainings: 3
Specialization: 5
Staff retention: 1
Stand-alone discipline: 2
Students: 1
Survey: 1
Taxonomy: 1
Teaching methodologies: 2
Technical research: 1
Training: 7
Undergraduate: 5
University of Florida: 1
University training: 19
Youth: 11
